# The role of macrophage polarization in tendon healing and therapeutic strategies: Insights from animal models

**DOI:** 10.3389/fbioe.2024.1366398

**Published:** 2024-02-29

**Authors:** Yicheng Wang, Xiao Lu, Jianxi Lu, Philippe Hernigou, Fangchun Jin

**Affiliations:** ^1^ Department of Pediatric Orthopedics, Xin Hua Hospital Affiliated to Shanghai Jiao Tong University School of Medicine, Shanghai, China; ^2^ Shanghai Bio-lu Biomaterials Co., Ltd., Shanghai, China; ^3^ Shanghai Technology Innovation Center of Orthopedic Biomaterials, Shanghai, China; ^4^ University Paris East, Orthopedic Hospital Geoffroy Saint Hilaire, Paris, France

**Keywords:** macrophage polarization, tendon healing, M1 and M2 macrophage, tissue repair, inflammatory response, therapeutic strategies, biomaterials in tendon repair, animal models

## Abstract

Tendon injuries, a common musculoskeletal issue, usually result in adhesions to the surrounding tissue, that will impact functional recovery. Macrophages, particularly through their M1 and M2 polarizations, play a pivotal role in the inflammatory and healing phases of tendon repair. In this review, we explore the role of macrophage polarization in tendon healing, focusing on insights from animal models. The review delves into the complex interplay of macrophages in tendon pathology, detailing how various macrophage phenotypes contribute to both healing and adhesion formation. It also explores the potential of modulating macrophage activity to enhance tendon repair and minimize adhesions. With advancements in understanding macrophage behavior and the development of innovative biomaterials, this review highlights promising therapeutic strategies for tendon injuries.

## 1 Introduction

Tendons, dense connective tissues responsible for transferring force from muscles to bones, play a crucial role in movement by storing elastic energy and withstanding immense tensile forces. (Docheva et al., 2015). Various injury mechanisms, such as acute overload, tearing, overuse, or age-related degeneration, can lead to tendon injuries. (Thomopoulos et al., 2015). Despite significant advancements in surgical and rehabilitation techniques, tendon repair may encounter postoperative complications. (Voleti et al., 2012). Tendon adhesion, a major complication following tendon injury, affects approximately 40% of patients after surgery, restricting tendon gliding ability and potentially leading to lifelong disability. (de Putter et al., 2012; Titan et al., 2019).

To understand tendon adhesion and healing, it is essential to firstly explore tendon biology, focusing on its collagen composition. Natural tendons are characterized by a sophisticated hierarchy of collagen intermingled with tenocytes and non-collagenous elements. (Benjamin et al., 2008). The tendon as a whole is wrapped in a thin layer called the epitenon. Beneath this layer are the fascicles, aligned with the tendon’s length and visible post-dissection, each surrounded by the endotenon, which also provides blood and nerve supply to the tendon. (Voleti et al., 2012).

Based on this structural foundation, we examine the detailed composition of tendon fascicles, crucial components of tendon architecture. Tendon fascicles consist of collagen fibers interspersed with tenocytes and are identifiable under optical microscopy at about 10 μm in diameter. (Screen et al., 2004). Electron microscopy further uncovers collagen fibrils, marked by periodic crimping in relaxed states. (Sharma and Maffulli, 2005). At a more minute level, microfibrils, formed from cross-linked tropocollagen molecules, lay the foundation of this structure, with tropocollagen being a water-soluble triple helix of polypeptide chains. (Screen et al., 2004). These complex structures, down to tropocollagen, are essential for understanding tendon response to stress and repair initiation.

The critical issue of tendon adhesion following injury or surgery was a key focus of current study. (Legrand et al., 2017). Further research has been dedicated to understanding the genesis and prevention of these adhesion, particularly in healing intrasynovial flexor tendons. (Voleti et al., 2012). Animal studies have shed light on critical elements influencing tendon repair and adhesion development, including initial injury severity, quality of surgical repair, and the significance of mechanical loading. (Thomopoulos et al., 2015). While mechanical loading facilitates collagen type III synthesis and boosts growth factor levels as well as cellular and matrix activities at the injury site, its excess can compromise healing. (Wong et al., 2009). Additionally, prolonged immobility is also implicated in adhesion development, as evidenced in various animal models. (Wong et al., 2014).

In the context of these findings, adhesion prevention has become a central goal in tendon repair research, especially considering its role in functional recovery, as adhesions can complicate the healing process and impair functional recovery. (Hu et al., 2023). In light of this, researchers have come to realize that the immune system plays an important role in tendon healing and adhesion formation, many studies have focused on how to modulate the immune response at the injured site. (Chisari et al., 2020). Bao et al. (Bao et al., 2024) expand the horizon of the anti-inflammatory effects primarily driven by sympathetic nerve through β2 adrenergic signals on macrophages, and made a history of using sympathetic stimulation to significantly prevent macrophage-mediated peritendious inflammation. In this context, the role of macrophages, particularly their involvement in the inflammatory response and modulation of the healing process, has garnered increasing attention. This focus stems from the understanding that inflammatory processes, driven largely by macrophages, are critical in the formation of adhesions during tendon healing. (Sunwoo et al., 2020). Additionally, researchers have recognized the significant impact of macrophages on tendon healing and adhesion formation, leading to a shift in focus towards understanding how macrophages specifically regulate tendon adhesion. (Xu et al., 2020) (Table 1).

**TABLE 1:** The effect of macrophage targeted therapies on the healing tendons in animal models.

Injured tissue	Intervention	Effect on macrophage	Effect on tendon healing
Murine Supraspinatus Tendons	CCR2 Knockout (CCR2KO)	Reduced macrophage infiltration	Improved biomechanical properties (higher load-to-failure and stiffness) Eliasberg et al. (2023)
Murine Achilles Tendons	Acute Achilles tenotomy and repair	A shift to M2 macrophages coordinating ECM deposition and tissue repair	Agent of degradation and repair in injured tendon tissue Sugg et al. (2014)
Murine Supraspinatus Tendons	Mechanical stimulation	Macrophage M2 polarization	Promoted MSCs chondrogenesis, and improved matrix formation Wang et al. (2023a)
Murine Flexor Digitorum Longus Tendons	Neutralization of active TGF-β1 and genetic manipulation	Reduced TGF-β1 production by macrophages	Attenuation of peritendinous adhesion formation Li et al. (2023)
Murine Flexor Digitorum Longus Tendons	COX siRNAs and Pla1a/Etv1 axis-related treatments	M2 macrophage polarization, increasing Pla1a protein secretion	Enhanced tendon healing Jing et al. (2023)
Murine Supraspinatus Tendons	polarized Macrophages and their derived exosomes	Exosome production and subsequent effects on FAPs	Reduced muscle atrophy and fatty infiltration Liu et al. (2023)
Murine Achilles Tendons	Extracellular vesicle-educated macrophages	M2-like immunophenotypic shift in EEMs	Improved mechanical properties of the healing tendon Chamberlain et al. (2019)
Murine Achilles Tendons	TDSCs seeded in Small Intestinal Submucosa scaffolds	Promotion of M2 macrophage polarization	Reduced adhesions and regulated ECM formation Mao et al. (2022)
Murine Achilles Tendons	Nano-micro fibrous woven scaffolds	Promotion of M2 macrophage polarization	Better tissue organization, reduced inflammatory response Cai et al. (2023)
Murine Achilles Tendons	Wnt3a-modified nanofiber scaffolds	Promotion of M2 macrophage polarization	Accelerated tendon healing, increased mechanical strength, reduced inflammatory response Wei et al. (2023)
Murine Achilles Tendons	Extracellular vesicles from inflammation-primed adipose-derived stem cells	Inhibiting M1 polarization and promoting an M1-to-M2 transition	Reduced inflammation, increased tendon cell proliferation, and improved collagen production Shen and Lane, (2023)
Murine Achilles Tendons	PDTC-loaded electrospun membranes	Inhibition of NF-κB pathway in macrophages	Reduced peritendinous adhesion and inflammation Lu et al. (2023)
Murine Peritendinous tissue	JSH-23-loaded PLA membranes	Inhibition of NF-κB phosphorylation and polarization	Tendon healing enhancement Wang et al. (2022)
Murine Achilles Tendons	Lipid nanoparticle-assisted miR29a delivery via core-shell nanofibers	Promotion of M2 macrophage polarization	Improved collagen composition and alignment, higher mechanical strength Chen et al. (2022)

The natural healing process of injured tendons involves three consecutive and overlapping stages: the inflammatory phase, proliferative phase, and remodeling phase. (Nichols et al., 2019). During the inflammatory phase, cytokines derived from platelets signal an elevation in vascular permeability, attracting circulating inflammatory cells, including phagocytic neutrophils, monocytes, and macrophages, to the injury site. (Marsolais et al., 2001; Chisari et al., 2020). The subsequent proliferative phase is characterized by the release of growth factors, such as vascular endothelial growth factor and members of the transforming growth factor beta (TGF-β) family, stimulating angiogenesis, granulation tissue formation, and fibroblast proliferation. (Wong et al., 2009). In the final remodeling phase, newly synthesized collagen fibers realign along the longitudinal axis of the tendon until they can withstand load. This process may take up to 2 years to complete. (Lomas et al., 2015).

In the context of macrophage-mediated regulation, the dominance of macrophages becomes significant beyond the initial 24-h period following injury. (Wong et al., 2009). Understanding how macrophages modulate the inflammatory and proliferative phases, and their potential impact on the subsequent remodeling phase, is essential for unraveling the intricacies of tendon healing. As we delve into the macrophage-specific aspects of tendon healing, the focus shifts towards strategies that enhance intrinsic healing while minimizing the impact of extrinsic healing, with the ultimate goal of improving the functional recovery of tendons (Stauber et al., 2020).

## 2 Pathology of macrophage-mediated adhesion

Macrophages are key components of the human innate immune system, widely distributed across connective tissues and various solid organs (Murray, 2017). Their high heterogeneity and plasticity enable them to play diverse roles in human diseases, driven by their ability to differentiate into distinct phenotypes under varying stimuli in the local microenvironment (Mosser and Edwards, 2008; Mould et al., 2019).

Classically activated macrophages, or M1 macrophages, emerge under the induction of lipopolysaccharide (LPS), interferon-gamma (IFN-γ), or tumor necrosis factor-alpha (TNF-α) (Di Benedetto et al., 2019). These M1 macrophages are known for their involvement in phagocytosis and display pro-inflammatory characteristics, essential in the body’s response to pathogens and injury.

Conversely, alternative activated macrophages, or M2 macrophages, develop in response to interleukin-4 (IL-4) or interleukin-13 (IL-13) (Viola et al., 2019). These M2 macrophages exhibit anti-inflammatory and pro-healing functions, playing a vital role in tissue repair and regeneration. Notably, M2 macrophages are further subclassified into M2a, M2b, M2c, and M2d subtypes, each characterized by unique activation stimuli, molecular expressions, and functional attributes (Paoli et al., 2014).

Furthermore, Lehner et al (Lehner et al., 2019) have identified a specific population of tissue-resident macrophages in murine and human tendons. These macrophages are key to phagocytosis, inflammatory cytokine secretion, and extracellular matrix-related proteins, playing a crucial role in tendon health and response to injury. Fujii et al. (2022) extend this understanding in the context of anterior cruciate ligament reconstruction (ACLR) in mice, identifying two distinct macrophage populations that infiltrate the tendon/bone interface post-surgery: the CD9^+^ IL1+ and CX3CR1+ CCR2+ macrophages. The CD9^+^ IL1+ macrophages peak 1 day after surgery with a highly inflammatory profile, transitioning later to a homeostatic state, while the CX3CR1+ CCR2+ macrophages accumulate more gradually and express interferon signature genes that might suppress bone formation. In addition, Li et al. (2023) provides the first evidence that macrophages are a primary source of TGF-β1, which is crucial in recruiting stem cell and make it differentiate into myofibroblasts to the surrounding site of injured tendon. This breakthrough sheds light on a crucial antiadhesion landmark of drug therapies, as it identifies an accurate cellular target to reduce peritendinous adhesion by manipulating the TGF-β1 pathway.

**FIGURE 1:**
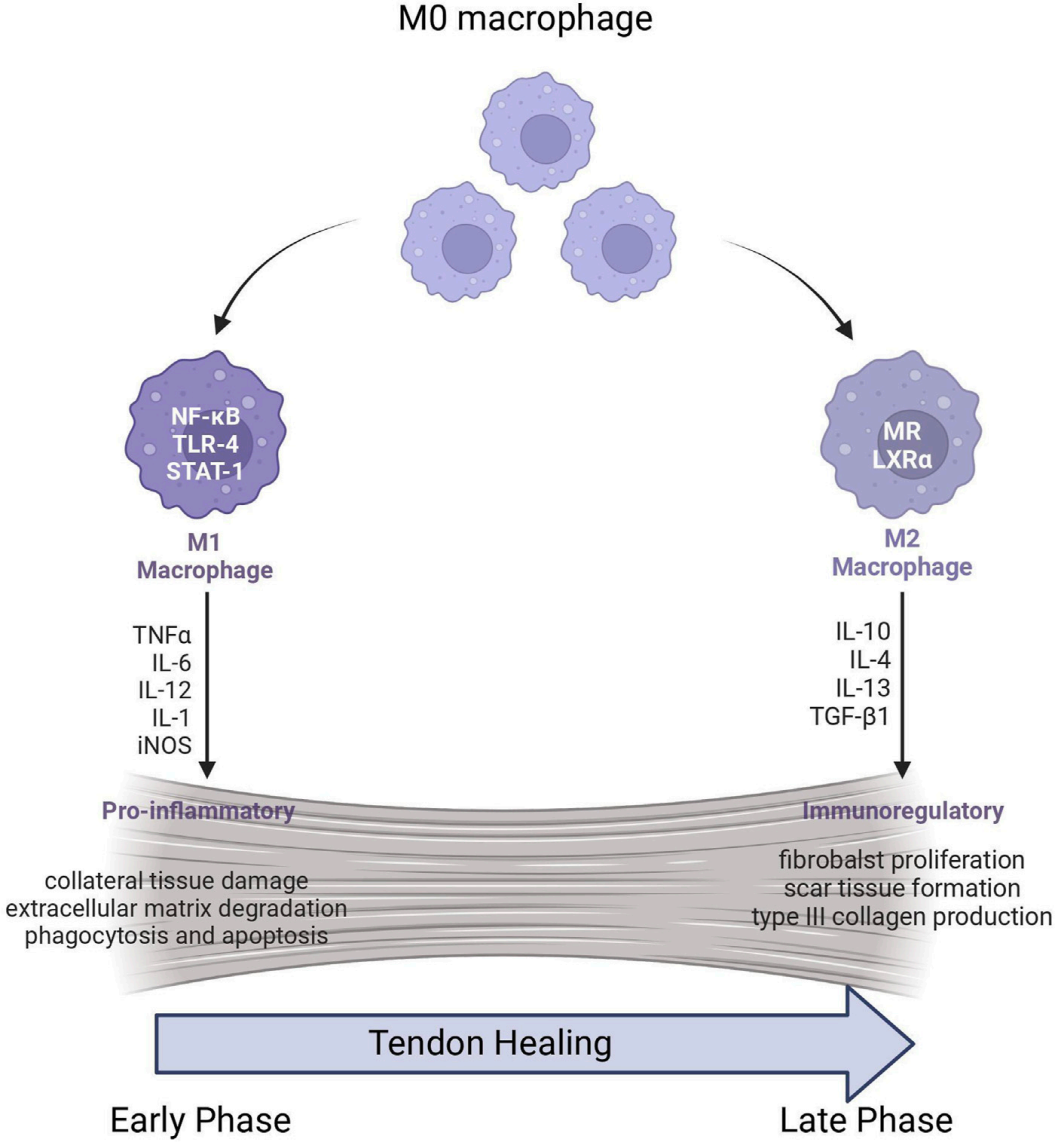
Macrophage polarization in Tendon healing.

In normal tendon tissue, both in the outer membrane and inner fibrous layers, macrophages are sparsely distributed. (de la Durantaye et al., 2014). However, this changes during the acute inflammatory phase following tendon injury. (Wong et al., 2009). In such instances, injured tendon tissue releases chemokines, including C-C chemokine ligand 2 (CCL2), which recruit immune cells, predominantly macrophages. (Sugg et al., 2014). This influx of macrophages leads to the further release of chemokines and cytokines, amplifying the inflammatory response and playing a crucial role in the initial phase of tendon healing. (Marsolais et al., 2001) (Figure 1).

The C-C chemokine receptor type 2 (CCR2) is particularly significant in this context, as it plays a vital role in the activation of macrophages, especially those exhibiting pro-inflammatory characteristics. (Liu et al., 2017). Utilizing CCR2 knockout models, researchers have demonstrated reduced recruitment of monocytes and a subsequent decrease in the inflammatory environment at wound healing sites. (Willenborg et al., 2012). This discovery has prompted exploration into CCR2 inhibition as a potential therapeutic approach, with studies in various conditions, including traumatic brain injury and nonalcoholic fatty liver disease, showing promising results. (Morganti et al., 2015; Flores-Toro et al., 2020). In tendon healing, particularly in the context of rotator cuff repair in CCR2 knockout mice models, there has been an observed decline in macrophage infiltration and suppression of interferon pathways. (Eliasberg et al., 2023).

Recent insights into macrophage-mediated adhesion pathology reveal the significant role of macrophage-secreted Secreted Phosphoprotein 1 (SPP1) in aggravating tendon adhesions. (Liu et al., 2022). Moreover, the interaction of SPP1 with fibroblasts, via cytokine secretion and cellular communication, leads to enhanced fibroblast activation and migration, further exacerbating adhesion formation. (Kapur et al., 2019). Wang et al. (Wang et al., 2024) further reveal that elevated SPP1 expression in macrophages enhanced fibroblasts activation to myofibroblasts through CD44 positive feedback pathway, which indicates a crucial cascade amplification between macrophages and myofibroblasts in the field of in inflammatory hyperplasia.

## 3 M1 macrophage-mediated adhesion mechanisms

In the early stages of tendon healing, the infiltrating macrophages at the site of injury are predominantly of the M1 phenotype (Sunwoo et al., 2020). Their concentration significantly increases within the first 2 weeks of tendon healing, and they localize to the newly formed tendon tissue and areas of tissue remodeling (Marsolais et al., 2001; Sugg et al., 2014). M1 macrophages contribute to the propagation of inflammatory responses by releasing a range of pro-inflammatory cytokines and mediators, such as interleukin-1 (IL-1), IL-6, IL-12, tumor necrosis factor-alpha (TNF-α), and reactive nitrogen and oxygen species (Barrientos et al., 2008; Koh and DiPietro, 2011). While they exhibit stronger microbicidal properties, M1 macrophages also have an increased potential for causing collateral damage to surrounding healthy tissues (Chamberlain et al., 2011; Sica and Mantovani, 2012). Additionally, M1 macrophages contribute to the degradation of the extracellular matrix, engaging in processes such as phagocytosis of cellular debris and apoptosis (Mosser and Edwards, 2008).

Given the pivotal role of M1 macrophages in the early stages of tendon healing, it is important to understand the underlying mechanisms that regulate their activity. One such critical aspect is epigenetic regulation, particularly through DNA methylation (Chen et al., 2023). Crucial DNA methyltransferases like DNA methyltransferase 3b (DNMT3b) and DNMT1 are involved in the polarization of these macrophages. DNMT3b inhibits peroxisome proliferator activated receptor (PPAR)γ1, a regulator of the anti-inflammatory M2 phenotype, thereby promoting the M1 phenotype crucial for the early inflammatory response in tendon healing (Yang et al., 2014). DNMT1 contributes by mediating the hypermethylation of genes that are essential for the pro-inflammatory activities of M1 macrophages (Denis et al., 2011). This intricate regulation of gene expression through epigenetic mechanisms underscores the complexity of M1 macrophage behavior in tendon healing and their critical role in initiating the inflammatory response that is essential for early stages of tissue repair.

Furthermore, the emerging role of non-coding RNAs (ncRNAs) in macrophage polarization is gaining attention (Ning and Liu, 2013). MicroRNAs (miRNAs) and other ncRNAs, such as long non-coding RNAs (lncRNAs) and circular RNAs (circRNAs), are significant regulators of macrophage behavior. For example, under the stimulation of IL-13 or TGF-β, miR-155 targets IL-13Rα1 and SMAD2, leading to a bias towards M1-like gene expression (Louafi et al., 2010). Beyond miRNAs, there is increasing interest in the role of long non-coding RNAs (lncRNAs) (Wang et al., 2020). LncRNA cyclooxygenase-2 (cox-2), for instance, is more prevalently expressed in LPS-induced M1 macrophages than in IL-4-induced M2 macrophages, and its suppression results in a decrease in M1 macrophage markers (Ye et al., 2018). Additionally, circular RNAs (circRNAs), which have a unique covalently closed loop structure, are also being studied for their relationship with macrophage polarization. Notably, circRNA Cdyl has been found to promote M1 polarization by inhibiting the nuclear translocation of interferon regulatory factor 4 (IRF4) (Song et al., 2022). In a similar vein, circRNA PPM1F is known to enhance the NF-κB signaling pathway following LPS stimulation, promoting M1 polarization (Zhang et al., 2020). This highlights the growing importance of understanding ncRNA-mediated regulation in macrophages, particularly in the context of their role in tissue healing and adhesion mechanisms.

Besides, an increasing number of studies indicate that M1 macrophages may influence the tissue microenvironment through the secretion of exosomes, which function to transport molecules containing biological information (Momen-Heravi et al., 2014). Lou et al. (2023) discovered that miRNA-155-5p, which is highly expressed in exosomes derived from M1-polarized macrophages, exerts antiangiogenic effects by targeting the GDF6-Akt axis, ultimately impacting the healing process in diabetic conditions.

## 4 M2 macrophage-mediated adhesion mechanisms

In contrast to M1 macrophages, M2 macrophages play a significant role in promoting fibroblast proliferation and stimulation of new tissue deposition. (Mantovani et al., 2002; Sun et al., 2023). The increase in the concentration of M2 macrophages primarily occurs in the later stages of the tendon healing process, especially in the region where the tendon extracellular matrix is located (Nichols et al., 2019). Sugg et al. (2014) found that in healing mouse tendons, the concentration of M2 macrophages within the first 28 days post-injury was similar to that of normal, uninjured tendon tissue. However, after 28 days of tendon injury, there was a significant increase in M2 macrophages, becoming the predominant macrophage phenotype at the site of injury. Wang L. et al. (2023) found that mechanical stimulation promotes the polarization of macrophages into the M2 phenotype and secretion of elevated levels of TGF-β1, ultimately, facilitates the chondrogenic differentiation of MSCs and enhances the process of tendon to bone healing in an acute rotator cuff repair model. Interestingly, while the study found that TGF-β1 secreted by M2 macrophages promotes tendon repair, another study revealed a potential drawback of TGF-β1, as it may contribute to tendon adhesion. The research findings by Li et al. (2023) suggest that TGF-β1 derived from M2 macrophages recruits mesenchymal stem cells and promotes the formation of myofibroblasts in tendon adhesion. Besides, due to its association with the inhibition of pro-inflammatory cytokines including IL-1β, IL-8, GM-CSF, and TNF-α, TGF-β1 is involved in terminating the inflammatory response during tendon healing, with M2 macrophages playing a role in this process (Fadok et al., 1998). Another research has been demonstrated that M2 macrophages facilitate tendon healing by secreting phospholipase A1 member A (Pla1a) (Jing et al., 2023). The secretion of Pla1a not only promotes tendon cell proliferation and reduces apoptosis but also leads to decreased Etv1 expression. This dual action of Pla1a, regulated by M2 macrophages, plays a critical role in reducing tendon adhesion and enhancing cell viability.

Contrasting with the earlier discussed role of M1 macrophage-derived exosomes, Liu et al (Liu et al., 2023) found that exosomes derived from M2 macrophages uniquely influence the differentiation of fibro-adipogenic progenitors, highlighting their distinct role in tendon healing mechanisms. The study provides detailed insights into the dynamic interactions between these exosomes and fibro-adipogenic progenitors, emphasizing that M2 macrophages significantly promote brown/beige fat differentiation, a crucial factor for effective muscle regeneration and reducing fatty infiltration.

M2 macrophage could also be associated with an elevated propensity for scar tissue formation. Wojciak et al (Wojciak and Crossan, 2008) found that the existence of inflammatory cells within the synovial sheath and epitenon during the healing process of tendons prompts synovial fibroblasts and epitenon cells to augment their fibronectin synthesis, thereby establishing a framework that facilitates the subsequent formation of adhesions. Moreover, an elevated M2 macrophage activity was detected in the fibrotic healing process observed in murine flexor digitorum longus tendons with Type II Diabetes (Ackerman et al., 2017). These fibrotic tendons displayed diminished biomechanical strength when compared to the repaired tendons of the nondiabetic control group. This excessive fibrosis could potentially be attributed to the excessive production of TGF-β1 by the M2 macrophages, which has been associated with the development of pathological fibrotic conditions in various tissues (Colwell et al., 2005).

In summary, the complex role of M2 macrophage is greatly due to the distinction between intrinsic and extrinsic healing processes in tendon repair. Intrinsic healing, originating from within the tendon, involves tenocytes and internal collagen synthesis, aiming for the restoration of normal tendon structure and function (Stauber et al., 2020). This contrasts with extrinsic healing, where repair is facilitated by external cells including fibroblasts and macrophages, often leading to the formation of adhesion (Voleti et al., 2012). Therefore, while M2 macrophages are integral to anti-inflammatory responses and promote tissue remodeling, which is beneficial in early stages of tendon repair, their prolonged predominance in later stages can inevitably promote extrinsic healing. Excessive M2 activity may lead to an imbalance in the healing process, deviating from the desired intrinsic repair pathway. Thus, in developing therapeutic strategies for tendon injuries, a critical goal is to modulate macrophage activity to encourage a balance that supports intrinsic healing, while mitigating the risk of excessive extrinsic tissue formation. This balance is essential for optimal tendon recovery, emphasizing the need for precise temporal and spatial control of macrophage phenotypes during the healing process.

## 5 Reprogramming of M1 to M2 transition

An increasing amount of evidence suggests that macrophages exhibit a vast spectrum of phenotypes and functions, shaped by specific differentiation signals, surrounding cell types, and the molecular context of different tissues. (Williams et al., 2018; Lehner et al., 2019). This diversity is more intricately understood through advanced technologies such as single-cell RNA-seq and single-cell mass cytometry by time of flight, which allow for the analysis of macrophage phenotypes with unprecedented resolution. (Murray, 2017; Arlauckas et al., 2021). These studies reveal that macrophages exist in a continuum of numerous subtypes, emphasizing the complexity of their roles in various physiological contexts. While the classification of macrophages into M1 and M2 phenotypes provides a useful framework, it serves primarily as a starting point for exploring the regulation of the optimal balance between inflammation and regeneration during tendon healing.

### 5.1 Interaction between MSCs and macrophages in M1 to M2 reprogramming

**FIGURE 2:**
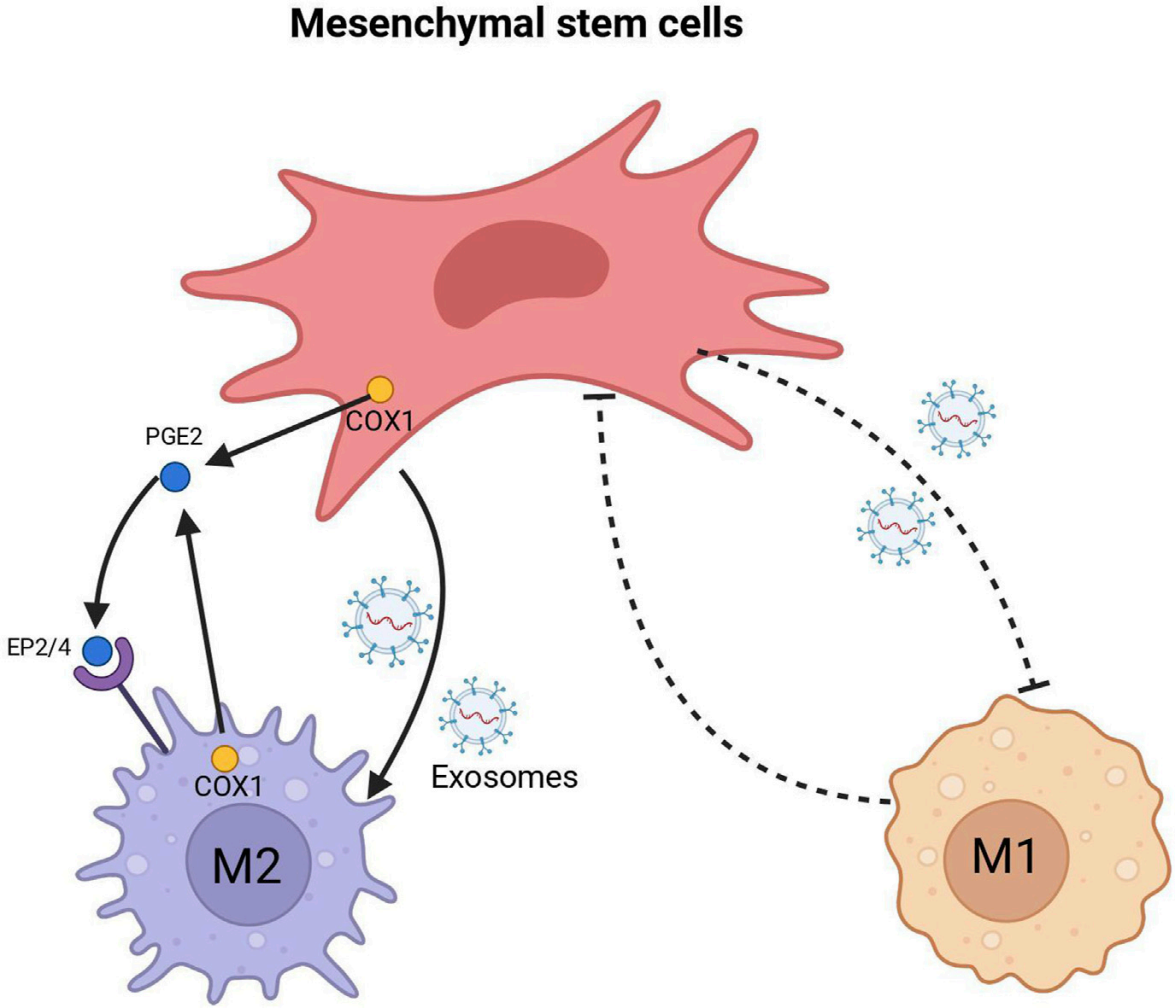
MSC interacts with Macrophage.

Delving into the mechanisms of macrophage transformation, particularly the reprogramming from the M1 to M2 transition, it is crucial to understand the dynamic interplay of macrophages within the healing environment. A significant aspect of this reprogramming is the interaction between macrophages and mesenchymal stromal/stem cells (MSCs), which has been shown to critically influence tendon healing (Maggini et al., 2010). MSCs modulate macrophage behavior by inhibiting M1 markers such as TNF-α and iNOS, and promoting M2 polarization, thereby resulting in improved tendon and ligament healing (Abumaree et al., 2013). A recent study revealed that MSCs facilitated the transition of monocytes into macrophages, heightened the response to microbial stimuli, shifted naive macrophages towards an M1 state, and simultaneously reduced the activity of already activated M1 macrophages while promoting M2 macrophage activation (Vasandan et al., 2016). Despite the current lack of complete understanding regarding the mechanisms underlying MSC-induced macrophage polarization at various stages, several studies have identified certain key factors. For instance, Németh et al. (2009) found that MSCs preconditioned with LPS or TNF-α can modify macrophage behavior through the release of prostaglandin E2 (PGE2), which interacts with macrophages through the EP2 and EP4 receptors. Another study by Chamberlain et al. (2019) showed that macrophage can be educated with extracellular vesicles (EVs) instead of direct coculture with MSCs, suggesting a paracrine-mediated mechanism by which MSCs polarize macrophages. Injured tendons treated with these EV-educated macrophages exhibited improved mechanical properties, reduced inflammation, and earlier angiogenesis, therefore result in superior tendon healing. He et al. (2019) demonstrated that exosomes derived from MSCs can drive macrophages towards M2 polarization. Depletion of MSC-derived exosomes resulted in a reduction in the M2 phenotype of macrophages, suggesting that MSC transplantation induces M2 polarization of macrophages and facilitates wound healing through the transfer of microRNAs within exosomes (Figure 2).

### 5.2 Comprehensive insights of biomaterials and mechanical stimuli in macrophage polarization

The pivotal role of biomaterials and scaffolds in directing macrophage polarization, a crucial aspect of tendon healing, cannot be overstated. Recent studies have highlighted that material cues can induce macrophage polarization towards either a pro-inflammatory or pro-resolving phenotype, which in turn leads to prolonged inflammation or tendon regeneration, respectively (Lin et al., 2018). (Hotchkiss et al., 2016) highlighted the significance of biomaterials’ chemical properties in this context, finding that different titanium-based surfaces affect macrophage activation. This study underscores the influence of surface properties on macrophage behavior and tissue remodeling. Wang et al. (2022) demonstrated that phosphorylation of NF-κB is an excellent unidirectional molecular switch to M1, which gives a insightful solutions to the long-standing challenge of selective control of macrophage polarization. Lu et al. (2023) inhibited M1 macrophages by NF-κB inhibitor PDTC to significantly reduce tendon adhesion formation and promote tendon healing, which firstly makes a record in peritendinous adapted treatment. Further, the physical structure of biomaterials plays a crucial role in macrophage phenotype modulation. Notably, the elongated shape of M2 macrophages compared to M1 macrophages has been leveraged to influence macrophage polarization (Tylek et al., 2020). McWhorter et al. (McWhorter et al., 2013) demonstrated that macrophage elongation, induced by aligned topography, leads to M2 polarization, a process inhibited by disrupting actin or myosin. Chen et al. (Chen et al., 2010) and Luu et al. (Luu et al., 2015) further explored this concept, observing maximal elongation and anti-inflammatory cytokine production in macrophages on substrates with 400–500 nm wide grooves.

The elasticity of substrates also impacts macrophage behavior, with studies showing that different stiffness levels affect activation and cytokine profiles. (Patel et al., 2012). Comparisons of 2D and 3D collagen matrices revealed that 3D environments are more conducive to pro-resolving cytokine secretion, suggesting their suitability for future studies in macrophage polarization. (Friedemann et al., 2017). The influence of mechanical loading on macrophage polarization during tendon healing is another crucial aspect. Blomgran et al. (Blomgran et al., 2016) found that mechanical loading delays the shift from M1 to M2 macrophages and Treg cells during tendon healing in rats by influencing the inflammatory response. This delay in macrophage polarization, caused by mechanical loading, potentially impacts the timing and quality of tendon repair. Conversely, Schoenenberger et al. (Schoenenberger et al., 2020) reported that mechanical loading tends to promote a shift toward an M2-like macrophage phenotype, considered beneficial for tissue healing. These insights highlight the need for further evaluation in biomaterial design to modulate macrophage polarization for improved tendon healing outcomes. Understanding the interplay among biomaterials, mechanical stimuli, and macrophage behavior is vital for advancing tendon repair strategies.

### 5.3 Exploring specific biomaterials in macrophage polarization and tendon healing

With the growing focus on manipulating the inflammatory response via biomaterials and scaffolds, considerable research has been directed towards developing various materials. These materials, each with unique attributes and mechanisms, are pivotal in influencing macrophage polarization, underlining their importance in tendon healing advancements. Small Intestinal Submucosa (SIS), as a naturally occurring decellularized matrix material, has been used to treat tendon defects in animals and has shown the ability to enhance tendon tissue regeneration. (Gilbert et al., 2007; Zhang et al., 2019). Mao et al. (Mao et al., 2022) seeded tendon-derived stem cells (TDSCs) onto a hydrogel coating of SIS to promote proliferation and enhance their adhesion and differentiation capabilities. In a 12-week rat Achilles tendon defect model, the combination of SIS scaffold and TDSCs promoted tendon regeneration and induced polarization of macrophages towards the M2 phenotype at the injured site, demonstrating their ability to modulate the immune micro-environment.

Derived from the natural macromolecule amniotic membrane, the decellularized amniotic membrane is another naturally derived biomaterials that has gained prominence due to its unique characteristics. (Tenenhaus, 2017). A recent study highlights the effectiveness of using decellularized amniotic membrane in tendon sheath repair to prevent adhesion. (Liu et al., 2018). This approach is marked by its ability to reduce inflammation and tissue swelling, as well as minimize adhesion formation. Additionally, the amniotic membrane group demonstrated enhanced biomechanical properties in the early postoperative phase compared to control groups. Studies have shown that biologically derived materials, such as decellularized surgical meshes, influence macrophage polarization, with a higher presence of M2 macrophages correlating with positive tissue remodeling outcomes. (Brown et al., 2012). Besides, functional biomaterials developed from naturally derived polysaccharides for tissue regeneration and pharmaceutical application have shown their role on altering macrophage phenotypes and influencing the immune response and tissue healing by recognizing cell membrane receptors. (Li and Bratlie, 2021). These findings suggest the potential of decellularized amniotic membrane as an effective biological material for tendon sheath reconstruction, contributing to improved healing and functionality while decreasing adhesion risks by modulating macrophage activity towards a constructive remodeling phenotype.

Recent trends in research have shown an increased focus on synthetic biomaterials over naturally derived ones. This shift reflects the versatile and customizable nature of synthetic materials, which offers broader possibilities for manipulating macrophage polarization in tendon healing. Cai et al. (Cai et al., 2023) developed a novel high-strength nano-micro fibrous woven scaffold with native-like anisotropic structure and immunoregulatory function for tendon tissue engineering application. This scaffold, made from polylactic acid and silk fibroin, effectively modulate the polarization of macrophages towards the M2 phenotype, demonstrating significant immunomodulatory capabilities. Additionally, research involving a Wnt3a-modified nanofiber scaffold, as demonstrated in a study, further underscores the potential of material-based approaches in modulating macrophage polarization. (Wei et al., 2023). This scaffold, designed to deliver the Wnt3a protein, not only facilitated the early functional recovery of Achilles tendon injuries in rats but also promoted the transition from an M1-dominated macrophage microenvironment to an M2-dominated one at the injury site, thus supporting tendon regeneration through an immunomodulatory mechanism. Similarly, a study by Shen et al. (Shen and Lane, 2023) revealed that extracellular vesicles from primed adipose-derived stem cells can effectively modulate the macrophage response towards M2 polarization, aiding in reducing inflammation and enhancing tendon healing, providing a complementary biological approach to the material-based strategies.

A notable advancement in tendon repair is highlighted in the work of Cai et al. (Cai et al., 2022), where the synergistic combination of self-healing hydrogel and siRNA nanoparticles presents a groundbreaking approach in macrophage modulation. This innovative design integrates the mechanical resilience and biocompatibility of hydrogels with the targeted gene-silencing capability of siRNA nanoparticles. Furthermore, the influence of biomaterial degradation products on macrophage behavior is a critical factor in peritendinous adhesion. (Wang S. et al., 2023). The findings suggest that the degradation of polylactide nanofibers could potentially modulate the inflammatory response and aid in tissue remodeling through the STAT6 signaling pathway. Their breakthrough of mechanism about Polylactic acid degradation related M2 polarization around tendon can address previously immunoreactivity challenging problems within the field of re-adhesion.

Naturally derived biomaterials such as Small Intestinal Submucosa (SIS) and decellularized amniotic membrane are known for their biocompatibility and anti-inflammatory properties, which are advantageous in reducing inflammation and adhesion in tendon healing. On the other hand, synthetic materials like polylactic acid and silk fibroin scaffolds are notable for their customizability and control over macrophage responses. While natural materials bring biological compatibility, synthetic alternatives offer tailored functionality, though they might require intricate engineering for optimal biocompatibility. This contrast emphasizes the need for careful material selection in tendon repair, based on specific therapeutic objectives.

### 5.4 The role of molecular pathway NF-κB in macrophage polarization

Furthermore, the understanding of macrophage polarization in tendon healing extends beyond material-based strategies to molecular mechanisms. In this realm, the role of NF-κB as a transcription factor is crucial. NF-κB plays a significant role in promoting the classical activation of macrophages, typically associated with the M1 phenotype. (Chen et al., 2017; Fan et al., 2020). Lu et al. (Lu et al., 2023) encapsulated the NF-κB inhibitor PDTC in electrospun polylactic acid (PLA) membranes, demonstrating that inhibiting NF-κB in macrophages can reduce tendon adhesion formation. Wang et al. (Wang et al., 2022) used the selective NF-κB inhibitor JSH-23 to demonstrate its role in macrophage polarization and the release of inflammatory cytokines. They confirmed that phosphorylation of NF-κB contributes to M1 polarization and the release of pro-inflammatory cytokines. Moreover, Chen et al. (Chen et al., 2022) loaded miR-29a into lipid nanoparticles incorporated into electrospun fiber membranes, finding that miR-29a downregulated NF-κB p65 expression and nuclear translocation at the injury site, promoting M2 polarization and inhibiting inflammation. These studies highlight the importance of targeting molecular pathways such as NF-κB to modulate macrophage behavior, providing insight into the complex interplay of cellular and molecular mechanisms in tendon healing.

However, it is crucial to maintain a balanced macrophage response during tendon healing. While promoting M2 polarization can suppress early inflammatory responses, excessive M2 activity may lead to adverse outcomes, such as the formation of adhesive tissues due to heightened fibroblast proliferation and excessive extracellular matrix deposition. (Colwell et al., 2005; Ackerman et al., 2017). Conversely, M1 macrophages, despite their potential for causing collateral tissue damage, are indispensable for effective tendon repair. Studies have shown that reducing M1 macrophages and neutrophils excessively does not improve Achilles tendon healing, whereas a moderate decrease in the M1/M2 ratio appears to be optimal for the healing process. (Chamberlain et al., 2011). This underscores the importance of a balanced M1 and M2 macrophage presence for optimal tendon recovery.

## 6 Conclusion

In conclusion, while this review has highlighted significant advancements in understanding macrophage polarization in tendon healing and adhesion mechanisms, the path forward calls for focused exploration. Future research should aim to unravel the complex molecular pathways influencing macrophage behavior, particularly the role of non-coding RNAs. Additionally, the development and clinical application of innovative biomaterials that can modulate macrophage activity presents a promising avenue for enhancing tendon repair and reducing adhesion formation. These focused areas of research hold the potential to significantly advance our understanding and treatment of tendon injuries.
